# Sperm Sociality: Cooperation, Altruism, and Spite

**DOI:** 10.1371/journal.pbio.0060130

**Published:** 2008-05-27

**Authors:** Tommaso Pizzari, Kevin R Foster

## Abstract

The idea of subfertile or altogether infertile sperm seems an evolutionary paradox, so why have they evolved in a diverse set of species, from molluscs to mice? Understanding sperm sociality may provide the answer.

A swimming sperm cell appears to perfectly capture the individualist Darwinian struggle, as it frantically races onwards towards a waiting egg. Consistent with this imagery, sperm morphology and behaviour in many organisms appears exquisitely designed to maximise the chances of fertilisation of each individual sperm cell [1]. However, there are numerous less obliging cases where sperm seem poorly suited to the task, even to the extent that the majority of sperm in an ejaculate may be infertile [2,3]. Why would such sperm evolve?

## Bringing Social Evolution to Sperm

The secret to unravelling the mystery of subfertile and infertile sperm may lie in understanding their social lives. Sperm evolution requires one to consider Darwinian selection on multiple interacting parties and at multiple levels, and this lends itself to the tools of sociobiology: kin selection and multi-level selection theory [4].

A male and female have just mated; what would one predict? Her evolutionary interests can be complex but, generally speaking, her priorities are to make sure that all of her eggs are fertilised, and that they are fertilised by sperm delivering the best genes for her offspring. It is in the interests of each individual sperm to rise to the challenge and do anything to fertilise an egg. This might mean a temporary alliance with some fellow sperm, but should others flounder and fail, all the better [5]. The male interests, however, are different. He has little to gain from sperm infighting, and instead only seeks to ensure that all of the eggs available are fertilised by his sperm. In other words, taking the perspective of the haploid genome in a sperm cell, different sperm haplotypes from the same male are in evolutionary conflict [5,6], while from the perspective of the diploid genome of the male parent, all sperm are equally valuable. This means that, in addition to conflict among individual sperm, there is also potential conflict between each sperm and the male, which could lead to an evolutionary arms race over which controls sperm morphology and behaviour [5,7].

**Figure 1:**
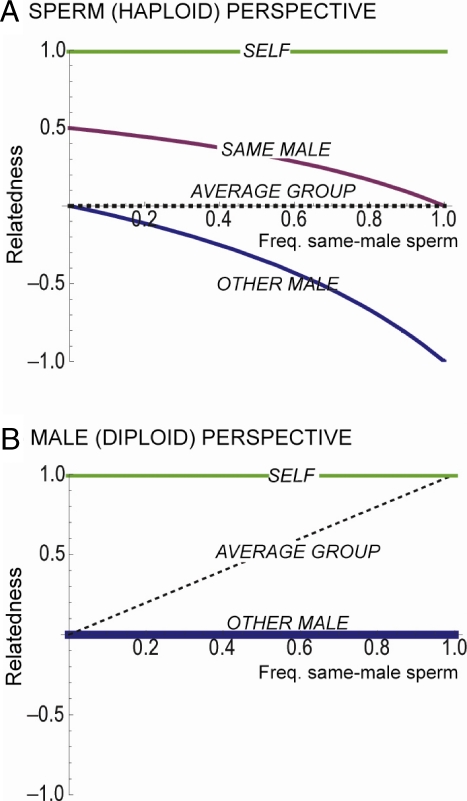
Genetic Relatedness among Sperm and Males as a Function of Female Re-Mating Rate (Risk of Sperm Competition) Social evolution theory predicts that relatedness is central to social behaviour. When two individuals share more genes in common than the population average, they are genetically related, and natural selection can favour altruistic behaviours that invest in another's reproduction, as with social insect workers. Formally, relatedness is calculated as (*p*_R_- *p*)/(*p*_A_ - *p*) where *p*_R_, *p*_A_, and p denote focal gene frequency in recipients, actors, and the population (Box 1, [42]). Calculations of relatedness require one to assign the relevant population scale at which individuals interact and compete (see Box 1, [16]). And, importantly, we are taking a different scale for the male and the sperm here: we assume that all evolutionary competition for sperm occurs within the female: she is the population for each sperm (Box 1). If the actions of sperm were to harm the female, there would also be competition among sperm in different females, which would change the relatedness values and, perhaps, evolutionary predictions [44]. (A) Sperm's perspective (population is at the scale of the female). If a female mates once, all sperm have the same probability of sharing genes, and relatedness at the scale of the female is zero. Adaptations that result from natural selection on sperm, therefore, are expected to favour the individual sperm's personal fitness interests. This may mean temporary alliances with other sperm, but may also mean strong competition among the sperm of the same ejaculate. If a female mates again, things change. The second male's sperm are less likely than average to share genes with the first (negative relatedness, Box 1), which can favour sperm that harm themselves just to reduce the chance that the other male's sperm fertilise eggs (spite). However, the mixing of sperm from competing males also means that a sperm cell is now more likely to share genes with sperm from the same male than with the average sperm present in the female (positive relatedness). This situation can favour altruism, and indeed, as the sperm of our focal male become rarer, altruism becomes a better option than spite (it is more difficult to knock-down a majority than support a minority). (B) Male perspective (population is at the scale of the real population). The only conflict for the male is with other males, and this conflict strengthens as the number of sperm inseminated by other males into the same female increases.

But now our female mates with a second male, and the battlegrounds shift somewhat. The two males are in strong conflict with one another as their ejaculates compete to fertilise the eggs, an inter-male process called sperm competition. This competition from a foreign male has important knock-on effects for the other conflicts. In particular, the presence of foreign sperm better aligns the evolutionary interests of each sperm and its male by increasing the incentive for cooperation with other same-male sperm (Figure 1). Pacts and alliances that would have been disadvantageous for a sperm cell in the absence of competing ejaculates suddenly make sense, and indeed, it is with sperm competition between the ejaculates of different males that we might expect the most elaborate sperm adaptations [1]. The potential for sperm to have a social life then seems clear, but does this help us to make sense of the diverse sperm behaviour seen in nature? We will argue here that it does.

**Box 1. Relatedness and the Scale of Competition**

“*He's not even the best drummer in the Beatles*.” Attributed to John Lennon, after a reporter commented that Ringo was not the best drummer in the world.

As for all assessments, assessments made in sociobiology must be tied to a specific reference scale, be it a local group or a global community [41,42]. This is nowhere more important than in the measure of genetic relatedness. In order to make social evolution predictions, relatedness should always be measured at the locus or loci in the genome that drive the social action of interest (average across-genome measures are only a proxy for the loci that drive a behaviour). Focusing then on an allele for, say, altruistic behaviour, one can ask whether a recipient of altruism has an above-average chance of having the allele that is present in the altruistic actor. That is, are the actor and recipient genetically related?

However, the reference to scale here is easily missed, as it is implicit in the need to define the probability of gene sharing above chance. Typically, “chance” is taken relative to the population frequency of the relevant alleles. For example, if there are two alleles at equal frequency in the population at a focal locus, then unrelated people will have ½ probability of having the same allele at that locus. Meanwhile, sisters will have a ¾ probability of allele sharing because, in addition to chance, they have a ½ probability of inheriting an identical allele from a parent. It is this 50% inflation relative to the average that gives the commonly cited ½ relatedness among siblings, which is why, evolutionarily speaking at least, you should be nice to your siblings. More formally, relatedness is calculated as (*p*_R_ - *p*)/(*p*_A_ - *p*) where *p*_R_, *p*_A_, and *p* denote focal gene frequency at a focal locus in recipients, actors, and the population at large [43].

Sometimes, however, it is informative to measure relatedness at different scales [41]. Consider, for example, two sisters queens in a honeybee colony. This is a rare occurrence because queens brutally fight until one is dead. But why would close relatives kill each other? The answer is simple: only one is needed to head the colony, and natural selection favours fighting to be the one that does. But it is also clear that the standard measure of relatedness does not usefully predict this behaviour: positive relatedness is not expected to drive fatal conflicts. This is fixed, however, by shifting scales. Honeybee queens are not fighting with all other queens in the population for their place in a colony (which would give relatedness of approximately 0.25, as queens are typically half-sisters). Instead, they fight only with the queens in their colony, which makes the colony the best reference scale. Taking two competing queens as an example, we can recalculate relatedness using the formula: *r* = (*p*_R_ - *p*)/(*p*_A_ - *p*) but where *p* (the “population” frequency) is no longer the frequency of the focal allele in the whole population of bees, but the average frequency in the two queens (local frequency). The queens are now less rather than more likely than chance (relative to the local frequency) to have the alleles in common with the other: the two queens are in fact negatively related [25,43]. (For example, taking a focal rare allele in a heterozygous queen, the focal individual frequency (*p*_A_) is 0.5, average frequency in the other queen (*p*_R_) is about 0.125 (¼ chance she is also heterozygous), and local frequency (*p*) is the average of the frequency in each, or 0.3125, giving *r* = (0.125 – 0.3125)/(0.5 – 0.3125) = −1). Just as positive relatedness predicts that there may be helping among individuals, so negative relatedness predicts that there may be harming. Natural selection favours queens that engage in a fatal duel, although the fighting is not spiteful but selfish because a queen's personal reproduction is increased if she wins (Table 1).

The key point then is that there is no single measure of genetic relatedness among individuals; rather it must always be set against a scale of reference [41]. Moreover, choosing the appropriate scale can help to capture the relevant biology. In the case of sperm in internally fertilising species, we expect the majority of competition among sperm to function within the female, and we therefore use the single female as the “population” measure *p* in Figure 1. Comparably, in externally fertilising species, sperm “populations” are represented by discrete spawning events. By contrast, males are competing at the scale of the real population, and therefore we measure relatedness among males with that scale of reference.

**Table 1:**
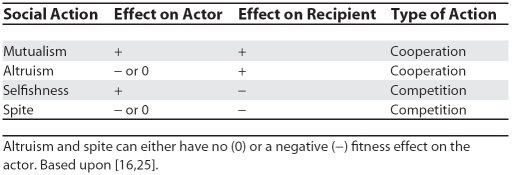
The Four Types of Social Action Based on Their Effect on the Direct Fitness (Lifetime Personal Reproduction) of the Actor and Recipient

First, we consider cases of sperm cooperation, where sperm have adapted to group together to mutual advantage, much like wolves that hunt in packs. We then turn to more paradoxical cases where the morphology and/or behaviour of a sperm cell actually reduces its probability of fertilisation, and interpret these traits in light of the evolution of altruism and spite (Table 1).

## Cooperation

A simple social action is to form some kind of team. Corporate life tells of its advantages: an effectively allied group will reliably trump a group in conflict. This principle of mutual cooperation is consistent with several sperm behaviours that appear to increase the fertilising probability of all team players.

*Sperm-grouping*: Sperm groups have been found across several vertebrate and invertebrate taxa, ranging from sperm pairs to massive aggregates containing hundreds of sperm [7]. These social sperm are often highly adapted to the task. The charming great-diving beetle Dytiscus marginalis has sperm with a distinctive flat side that allows some sperm to pair up—stuck together by the head—and use both tails to propel themselves onward [8]. Meanwhile, sperm of gyrinid beetles are attached via a third-party rod-like object produced in the male epididymis, the spermatostyle. This appears to facilitate and synchronise sperm migration to the female sperm storage organs, where the spermatostyle disintegrates, releasing the sperm [8].

**Figure 2:**
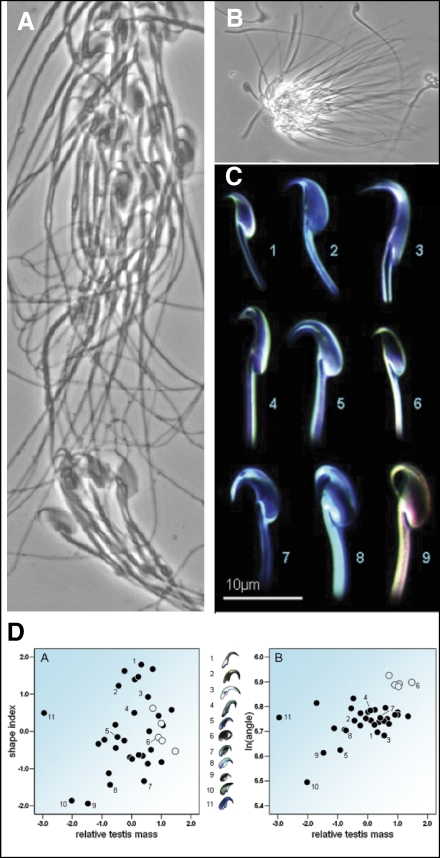
Sperm Trains in Rodents (A) Wood mouse A. sylvaticus sperm train where sperm are attached hook-to-hook or hook-to-flagellum (credit: Harry Moore). (B) Motile grouping of wood mouse sperm (credit: Harry Moore). (C) Apical hook morphology across different species of rodents (1, Bunomys fratrorum; 2, M. musculus; 3, R. norvegicus; 4, Dasymys incomtus; 5, Pseudomys oralis; 6, Maxomys surifer; 7, Melomys burtoni; 8, A. sylvaticus; 9, A. speciosus). From [10]. (D) The shape (left graph) and curvature (right graph) of the apical hook across different species of murid rodents in relation to the level of sperm competition (relative testes mass). From [10].

**Figure 3:**
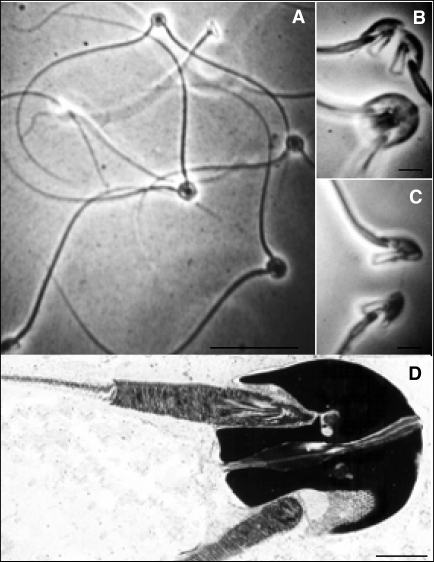
Conjugate Sperm Pairs in American Opossums (A) Paired and single sperm of the short-tailed opossum Monodelphis domestica. (B) Pairs of conjugate sperm attached by the heads, the top pair starting to separate after capacitation. (C) Pair of conjugate sperm separating. (D) Electron microscopy of exquisite sperm head alignment in conjugate sperm pair (credit: Harry Moore).

As might be expected, sperm grouping is sometimes found to drive more efficient migration towards the egg. Hundreds of sperm agglutinate by the head in the fishfly, Parachauliodes japonicus, and swim into the female spermatheca to the tune of a synchronised tail-beat, which propels them faster in large groups than in small [9]. More spectacularly social sperm are found in the humble Norway rat, Rattus norvegicus, and several other murid rodents, which have sperm with a distinctive hook-shaped head (Figure 2). Puzzling at first, this hook is now thought to help sperm to reversibly form groups of up to several hundred sperm (Figure 2A and 2B). And while no speed advantage in grouping was found in the house mouse, Mus musculus [10], sperm groups swim faster than single sperm in the Norway rat [10] and the wood mouse, Apodemus sylvaticus (discussed below) [3]. Sperm of the short-beaked echidna, Tachyglossus aculeatus, also form large groups of up to 100 in which coiled sperm heads are stacked tightly and cemented together, which again improves swimming speed [11]. Finally, in most species of American opossums, sperm rotate to align their head in pairs as they mature in the epididymis, leading to pairs of sperm conjugated by the head and propelled by the coordinated beat of both tails, which results in both a faster and straighter trajectory than solitary swimming sperm [12,13] (Figure 3).

Why it is good to swim more quickly? One reason might be that it minimises the time that sperm have to survive in a potentially hostile female environment. Perhaps the key driver for increased motility, however, is sperm competition between the ejaculates of different males. At least, there is growing indirect evidence of a link between sperm competition and social grouping. Across murid rodents, those species with relatively larger testes (a predictor of the level of sperm competition experienced by a species) tend to produce sperm with more pronounced apical hooks [10] (Figure 2C and 2D), which presumably promotes grouping. Short-beaked echidnas may also experience intense inter-male sperm competition because males have large testes and form queues of up to 11 individuals competing over the same receptive female [11]. Similarly, carabid beetle species with more complex male genitalia and longer periods of mate guarding, characteristics typical of species with a high risk of inter-male sperm competition, tend to have relatively large sperm bundles, suggesting that sperm competition promotes the evolution of larger sperm groups [14].

Are these effects due to natural selection acting on the male or on the individual sperm? The simplest explanation would be to look to the male, because the intensity of evolutionary competition he experiences is expected to scale with the degree of female promiscuity (dotted line, Figure 1B). In other words, if females only mate once, there is no sperm competition from a male's perspective, which may reduce his benefits from the formation of competitive sperm groups that swim against each other. By contrast, for the simplest case of random mixing among sperm in the female, the incentive for individual sperm to engage in competitive behaviours is expected to be high irrespective of female mating behaviour (dotted line, Figure 1A). This is because each sperm can benefit from out-swimming another sperm, regardless of whether that sperm comes from the same male or a different male. Or in the terminology of social evolution, sperm are always unrelated at any female re-mating frequency (dotted line, Figure 1A) if we measure average genetic relatedness among sperm at the scale of the female (see Box 1). Taking this simple sperm's-eye view of the world then, when sperm group randomly and grouping helps them compete, sperm are expected to group equally regardless of female promiscuity, which does not fit the data.

It would seem then that the observed link between sperm competition and grouping behaviour is all about the male. Or is it? There is another interpretation that puts the sperm back in the spotlight: perhaps sperm are able to specifically target and group together with their closest relatives. This might occur either through direct recognition of the same genotypes in other sperm [15,16], or more simply through a proxy that allows joining with same-male rather than foreign sperm. With non-random sperm mixing, natural selection may favour sperm that act altruistically and help related sperm at a fitness cost to themselves. And all else being equal, selection for altruistic grouping behaviours will increase with increased levels of mixing with the sperm of other males, which offers an alternative explanation for the observed link between inter-male sperm competition and grouping (purple line, Figure 1A). But do sperm preferentially group with others from the same male, and moreover, is there evidence for sperm altruism? With these questions, we return to the paradox of those sperm that cannot, or will not, fertilise an egg.

## Altruism

The rise of sociobiology in the sixties and seventies was largely driven by the problem of altruism: why does a honeybee worker, for example, sacrifice her personal reproduction to help queen and colony? The answer is a mixture of kinship—passing on shared genes through relatives—and coercion—insect workers are born subfertile and have their reproduction policed by other colony members [17]. With increased understanding of the evolutionary processes that drive altruism comes an increased appreciation that altruism has the potential to occur at all levels of biological organisation, including, of course, among sperm.

*Sperm trains in the wood mouse and conjugate opossum sperm*: As discussed above, sperm of the wood mouse attach themselves to each other by bending the apical hook on their head around the flagellum or the hook of another sperm, forming trains of hundreds of sperm that allow them to swim faster (Figure 2A and 2B) [3]. But swimming in a train also costs some sperm dearly. In order to fuse with an egg, mammalian sperm must undergo an “acrosome reaction”, in which their acrosome tip is bared of its membrane and sperm hydrolytic enzymes are released. When this happens near the egg, the acrosome reaction promotes fertilisation, but a premature reaction leaves sperm impotent and useless by the time they reach the egg. In the wood mouse, over 50% of the sperm forming a train undergo a premature acrosome reaction that prevents them from fertilising [3]. A comparable effect is seen in the opossum. As opossum conjugate sperm pairs move through the female oviduct and approach the egg, they split up, and while one swims on with fervour, the other falls away and loses motility [12,13].

Are these then examples of altruistic helping like that seen in social insect workers? This is not yet clear. What may be going on is a life-or-death lottery that carries extremely good odds. Take the opossum pairing. If sperm are all equally likely to be crippled, and pairing more than doubles their chances of fertilisation, it is in each sperm's personal fitness interest to buy a ticket [15]. In spite of the potential costs, therefore, train formation and conjugation may represent purely mutualistic behaviours (Table 1). It is notable, however, that these sperm groups form either before or shortly after ejaculation. This means that sperm will probably join with sperm from the same male rather than sperm from another male that mates before or afterwards; i.e., there may often be positive relatedness within the sperm groups (Figure 1, Box 1). When interacting with relatives, a sperm has the opportunity to transmit its genes by helping other sperm that carry them (indirect fitness) as well as by personally fertilising an egg (direct fitness) [16].

This opportunity paves the way, in evolutionary terms, for behaviours that reduce an individual sperm's chance of fertilising in order to increase the chances of another: sperm altruism. But what would altruism look like in these groups? One possibility is that some sperm group in circumstances that predictably lead to their own impotence, such as joining as pushers whose sole function is to help others to reach the egg ahead of the sperm from another male. Of course, it is also possible that sperm are simply forced to group by the male, which would mean that any sperm “altruism” is illusory in the sense that it did not evolve through natural selection acting at the level of sperm (sperm lack evolutionary agency). But given that the attachment phase seems to require autonomous sperm behaviour [3], it seems likely that both male and sperm interests effect the grouping. As in eusocial insects [17], therefore, a combination of kinship and coercion may work in concert to produce sperm sociality.

*Sperm heteromorphism*: The analogy with an insect worker caste appears to go even further in species with morphologically distinct sperm types, a phenomenon known as sperm heteromorphism. Typically, only one sperm type (eusperm) is involved in fertilisation, while the other type(s) (parasperm) do not or cannot fertilise the egg. One interpretation is that these sperm are simply developmental failures, but their sheer numbers, more than half of the sperm of an ejaculate in some cases, suggest otherwise [2]. In support of this theory, recent artificial insemination experiments in the silkworm moth Bombyx mori revealed that parasperm may facilitate transport of eusperm to the site of sperm storage and/or fertilisation [18], and the enormous size of the parasperm in some molluscs, up to 140 times larger than eusperm, may also help to shuttle eusperm to the eggs [19]. In other molluscs (e.g., Aporrhais pespelecanis), parasperm morphology suggests that they deliver nutrients either to eusperm or to the female [19], which is likely, either directly or indirectly via the female, to increase eusperm fitness. Finally, parasperm may sometimes reduce the spermicidal effects in the female reproductive tract, thereby saving some eusperm [20,21].

Phylogenetic studies suggest that sperm competition may be associated with the evolution of heterospermy in some taxa [22], but not in others [23]. And as for sperm grouping in mammals, it is currently unclear whether these behaviours constitute altruism on the part of the sperm, manipulation of sperm by the male, or some mixture of the two. A fuller understanding of the biology of these systems is required [24]. The important open questions for the sociobiology of parasperm are (again): how often do sperm from different males meet in a female? Can parasperm direct their actions based upon genetic relatedness? And, developmentally, are sperm able to influence whether they become a parasperm or a eusperm?

In most species, the extent to which the fate of a spermatid is determined by the male parent or by its own haploid genome is unclear. In some extreme cases, however, we already know the answer. For example, male butterflies produce two types of sperm: eupyrene and apyrene sperm. Apyrene sperm are parasperm that lack DNA altogether, which makes them simply an accessory of the male, as is the case for seminal fluids. In this case, there can be no male–sperm conflict, and sperm evolution is driven by the male alone. The incentive for producing apyrene sperm, however, appears still to result from evolutionary conflict; in this case with other males. Apyrene sperm in the green-veined butterfly, Pieris napi, have been shown to reduce the probability that a female will re-mate with another male [24]. Although the mode of action of these sperm is unclear, they are extremely motile and have been suggested to act as a “filler” that evolved to prevent the female from re-mating by stimulating her sperm storage organ and making it feel full.

## Spite

A more malicious and mysterious social behaviour is spite, whereby an actor reduces their personal fitness to harm a recipient [25]. Where there is the potential for altruistically helping close relatives, the potential to spitefully harm others naturally follows. The magic ingredient for spite is negative relatedness, whereby individuals have a less than average chance of sharing genes. As our simple analysis shows (Figure 1), negative relatedness between sperm abounds within the sperm storage organ of a promiscuous female, in which sperm of multiple males mingle, setting the scene for spite. The idea that sperm harm one another dates back 25 years in the empirical literature [26–29]. But is this really spite?

**Figure 4:**
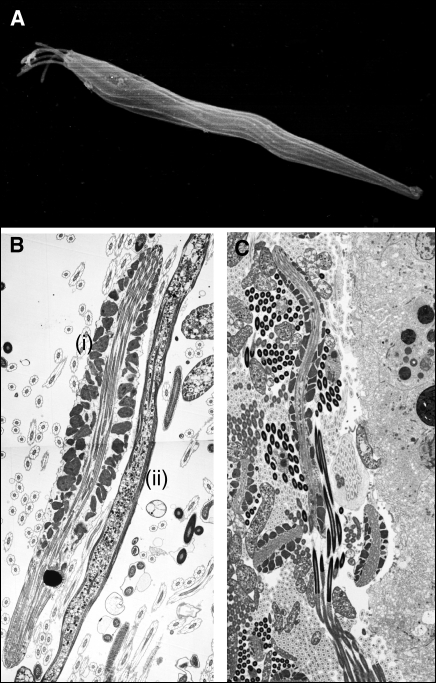
Mollusc Parasperm (A) Immature Oregon triton (Fusitriton oregonensis) lancet parasperm seen with scanning electron microscopy, showing the tail brush still present, which later develops into part of the body of the parasperm. (B) Montage of side-by-side transmission electron microscopy sections of the carrier (i) and lancet (ii) parasperm. (C) Montage of two transmission electron microscopy sections of a carrier parasperm transporting eusperm (long dark nuclei) with some cross-sections of eusperm and carrier and lancet parasperm (credit: John Buckland-Nicks).

Not always. Some examples are better interpreted in terms of selfishness by the male, such as sperm flushing. Insects such as the cowpea beetle, Callosobruchus maculatus, inseminate more sperm than the female can actually store [30]. This excess sperm cannot contribute to fertilisation but appears to flush out previously stored sperm from a competing ejaculate, and while this means that some sperm are harming others, these sperm do not seem to have much choice in the matter. But in other species, there may be a case for spitefully suicidal sperm. Human sperm were famously suggested to be heteromorphic, comprising one type that contributed to fertilisation and another, the kamikaze sperm, that sacrificed its own chances of fertilisation to neutralise the sperm of competing ejaculates, for example by blocking, incapacitating, or killing rival sperm [28]. While subsequent studies did not support this idea in humans [31,32], similar mechanisms might occur in some snails. For example, the Oregon triton, Fusitriton oregonensis, has two distinct parasperm types: sperm shuttlers (above) and lancets (Figure 4A–4C), and experiments that add a homogenate of parasperm to eusperm find that this causes the eusperm to clump together in vitro, an effect not seen when adding eusperm to eusperm [19]. Parasperm may also actively release compounds that harm eusperm: immature lancet paraspermatids are known to produce digestive enzymes that may end up being released in the female [19]. To the extent that these behaviours are caused by the sperm rather than the male, these observations are consistent with spiteful behaviours that evolved to harm the eusperm of other males.

## Future Directions

If we are to fully unravel the mystery of infertile sperm, we need a greater understanding of the evolutionary costs and benefits of sperm actions, and the extent to which sperm control their own fate rather than being forced by the male into behaviours that only appear altruistic or spiteful. The question of sperm autonomy lies in the relative degree to which sperm can express their genes when in the haploid state. There are clearly some constraints: mature sperm DNA is condensed, which limits its potential for expression [33,34]. However, there is also evidence for gene expression in sperm during and after meiosis [35,36], including the striking examples of segregation distorters. Segregation distorters are groups of linked genes that are able to prevent the proper development of sperm that lack them [37–39], such that in a heterozygote male, one half of the sperm will eliminate the other half. Here, evolutionary conflict is not strictly between the sperm and the male, but rather between the selfish segregation distorter linkage group and everyone else (all other genes in the sperm and the male). Conflict between sperm and male genomes may often be more subtle, and understanding its full scope and effects will benefit from a number of complementary approaches

Transcriptomic and mutant studies of sperm will help to reveal their potential to act autonomously and affect one another in an ejaculate, particularly when applied to different sperm haplotypes produced by heterozygous males [37]. An associated challenge is to understand whether sperm from different males segregate in space and time within multiply-mated females. At the extremes, sperm could be kept entirely separate or mix fully. An intermediate case, however, seems more likely; experimental studies using labelled sperm indicate that different ejaculates can stratify within the female's sperm-storage organs [40]. This case is also the most interesting, because it generates non-zero relatednesses that may select for complex social traits (see Box 1). An associated question is whether stratified sperm are able to actively recognise other sperm, or whether associations result from passive processes such as the spatial separation of ejaculates within a female. Further experiments that mix differentially labelled sperm would provide opportunities to test the potential for sperm kin recognition and sperm–sperm interactions in general.

The power of interspecies comparisons for our understanding of sperm biology is already clear from the link between sperm morphology and promiscuity (Figure 2). Another interesting comparison can be made among species with different genetic systems, in particular diploid and haplodiploid species (such as Hymenoptera). Haplodiploid females are diploid, but the males are haploid with clonal sperm that should lack the evolutionary conflicts seen in diploid males, both among sperm and between each sperm and the male [1]. Finally, we must better understand how the insemination of different ejaculates affects female fitness. Our simple relatedness calculations assume that sperm social behaviours have no negative effects on females or on the probability that eggs are left unfertilised (Figure 1, Box 1). If these assumptions are incorrect, we would need to consider an additional level of selection generated by competition among sperm populations inseminated in different females.

## The Sociobiology of Sperm

The ability of sperm to express their own genes suggests that we should not view them simply as passive automata that serve the male, but rather as semi-independent agents with their own evolutionary interests. And with this perspective comes the potential for true sperm sociality. As we have seen, sperm can benefit from joining forces with others, helping their kin, or even harming others. Whether these behaviours are formally altruistic or spiteful, however, remains to be seen.

What is clear is that the sperm–male partnership can be an uneasy one, steeped in potential conflicts. But like all partnerships, they will perform the best in the face of their worst foe: the sperm of another male that threatens to eradicate their genetic trace altogether. It is here that we can expect sperm to be the most social; here they will diversify in form and function, engaging one another in competition or coalition to satisfy those selfish genes. Appreciating this sociality is a promising step forward in unravelling the mystery of subfertile or sterile sperm. Conversely, sperm sociality represents fertile—but so far little explored—ground for the study of social evolution.
